# Genomic and Epidemiological Characterization of DENV‐1 and DENV‐2 Co‐Circulation During the 2023–2024 Dengue Epidemic in Espírito Santo, Brazil

**DOI:** 10.1002/jmv.71078

**Published:** 2026-07-30

**Authors:** Nicolli Ribeiro de Jesus, Caio Santos de Souza, Mariene R. Amorim, João Paulo Cola, Raquel Gomes Catozo, Bruno Luiz M. Guedes, Bruna Caetano Pimenta, Julia Sthefany Nunes Zordan, Yasmim Barcellos Madeira Rosa, Henrique Tamanini Silva Moschen, Aline Souza Areias, Tatiane Comerio, Isabela Valim Sarmento, Julia Miranda Fardin, Daniel Claudio de Oliviera Gomes, Kiven Kumar, Eng Eong Ooi, Camila Malta Romano, Creuza Rachel Vicente

**Affiliations:** ^1^ Post‐Graduate Program in Infectious Diseases Federal University of Espírito Santo Vitória Brazil; ^2^ Institute of Tropical Medicine University of São Paulo São Paulo Brazil; ^3^ Special Epidemiological Surveillance Unit Health Department of Espírito Santo State Vitória Brazil; ^4^ School of Biology Federal University of Espírito Santo Vitória Brazil; ^5^ Epidemiological Surveillance Service Health Department of Vitória Vitória Brazil; ^6^ Nucleus of Infectious Diseases Federal University of Espírito Santo Vitória Brazil; ^7^ Program in Emerging Infectious Diseases Duke‐NUS Medical School Singapore Singapore

**Keywords:** dengue virus, DENV‐1, DENV‐2, genomic surveillance, molecular epidemiology, phylogenetics

## Abstract

In 2023 and 2024, Espírito Santo state, Brazil, experienced its largest dengue epidemic in 30 years, with record numbers of cases and deaths. This study aimed to characterize confirmed dengue cases and the dengue virus (DENV) during the epidemic and pre‐epidemic periods. A population‐based descriptive study integrated with cross‐sectional genomic sampling was conducted using data from the eSUS Health Surveillance System (2020–2024) and molecular analysis of serum samples (2023–2024). Whole 26 DENV genomes were obtained and combined with public data for phylogenetic reconstruction. In 2023, 127 070 dengue cases were reported; 2.8% showed warning signs or severe dengue, 2.6% required hospitalization, and 99 cases resulted in death. In 2024, 131 170 cases were reported, with 2.0% showing warning signs or severe dengue, 2.3% requiring hospitalization, and 42 fatalities. Four lineages were detected: DENV‐1 genotype V lineages 1V_A and 1V_E.1, DENV‐2 genotype II lineage 2II_F.1.1.2, and genotype III lineage 2III_C.1.1. These lineages were closely related to viruses from other regions in Brazil and South America. Our findings highlight the complex dynamics of DENV in Espírito Santo state and hypothesize that the multiple introduced DENV lineages and genetic diversity may have contributed to the magnitude and sustained transmission observed over consecutive years.

## 1 Introduction

Dengue epidemics have increased in frequency and severity in Brazil in the period following the COVID‐19 pandemic, which substantially affected public health systems [1]. In 2023, the country reported more than 1.6 million cases and 1179 deaths, and the number of reports increased significantly in 2024, exceeding 6.6 million cases and resulting in 6041 confirmed deaths [2].

Several factors may have contributed to this increase, including the failure to control the *Aedes aegypti* vector, a situation exacerbated by the *El Niño* climatic phenomenon. Higher temperatures can accelerate the mosquito life cycle, increase its activity, and shorten the viral incubation period in the vector [3, 4]. Additionally, the constant reintroduction of the dengue virus (DENV) into the population is critical for sustaining epidemic cycles [5]. DENV comprises four genetically and antigenically distinct viruses (also referred to as serotypes) (DENV‐1, DENV‐2, DENV‐3, and DENV‐4) [6]. Each virus comprises different genotypes, genetic variants that may differ in transmissibility, virulence, and immune evasion capabilities [6]. These differences influence the magnitude and severity of epidemics [5]. Moreover, secondary heterologous infections are associated with increased disease severity through antibody‐dependent enhancement [6].

Although most DENV infections are asymptomatic or present with mild symptoms, such as fever, headache, retroorbital pain, myalgia, arthralgia, and rash, epidemics often lead to a higher number of cases with warning signs, severe dengue, and deaths. These outcomes are associated with increased plasma leakage, hemorrhage, or organ dysfunction and represent a significant burden to the public health system [7].

Espírito Santo state was one of the states most affected by dengue epidemics in Brazil in recent years, with over 190 000 suspected cases and 99 deaths in 2023, and more than 250 000 suspected cases and 42 deaths in 2024 [8]. Dengue has been endemic in the state since 1995, with other relevant epidemics occurring in 2011 (DENV‐1/DENV‐2; 55 017 suspected cases), 2013 (DENV‐4/DENV‐1; 83 008 suspected cases), 2016 (DENV‐1; 55 221 suspected cases), and 2019 (DENV‐2/DENV‐1; 81 451 suspected cases) [9, 10]. These recurrent epidemics highlight the sustained vulnerability of the population to different DENV types over time.

Both Brazil and Espírito Santo state have experienced dengue outbreaks in consecutive years in 2023 and 2024, a pattern that is unusual given the typical dengue epidemic cycle of 3–5 years. Years following major dengue outbreaks typically show low incidence, as a substantial proportion of the population becomes immune to the most prevalent DENV type [11]. That dengue outbreaks occurred in 2 consecutive years in Espírito Santo state is thus intriguing, and the underlying determinants remain unclear.

Given the unprecedented scale and mortality of the epidemics in Espírito Santo state in 2023 and 2024, and the possibility that DENV genetic factors may have contributed to the observed epidemiological outcome [12], this study aimed to characterize confirmed dengue cases and circulating DENV strains during the epidemic and pre‐epidemic periods. Using integrated epidemiological and genomic data, we identified multiple introductions of DENV‐1 genotype V, lineage 1V_E.1, and further report the introduction and expansion of the DENV‐2 Cosmopolitan genotype, lineage 2II_F.1.1.2, during the outbreaks in the state. These findings underscore the critical role of genomic surveillance in tracking viral diversity and detecting emerging lineages, contributing to improved preparedness for future dengue epidemics.

## 2 Materials and Methods

### 2.1 Approval of the Institutional Review Board

The Research Ethics Committee of the Health Science Center at the Federal University of Espírito Santo approved the research protocol under opinions 5.994.874 and 6.241.070. The study adhered to the ethical principles of the Declaration of Helsinki. All participants who collected a blood sample signed an informed consent form.

### 2.2 Study Design

A population‐based descriptive study integrated with cross‐sectional genomic sampling was conducted to characterize dengue epidemics in Espírito Santo state in 2023 and 2024 and to define the phylogeny of DENV detected during this period. The study also characterized the pre‐epidemic period after the last epidemic occurred in Espírito Santo state in 2019 [9].

### 2.3 Study Area

**Figure 1:**
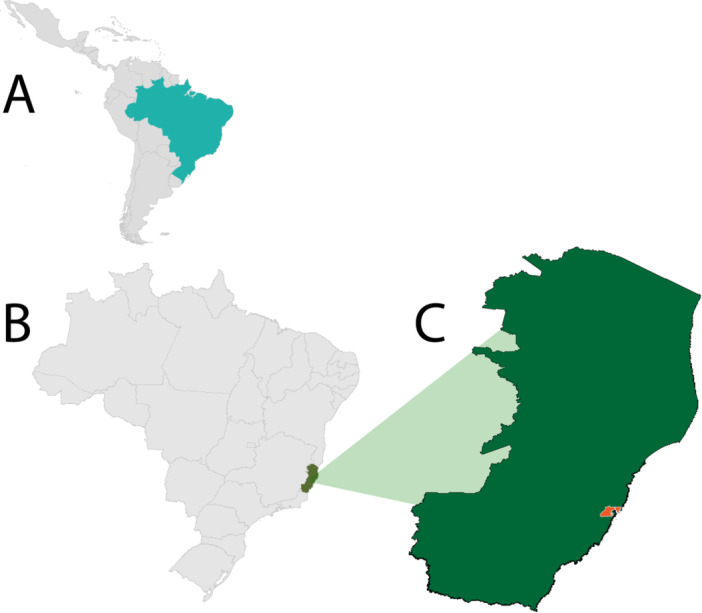
Map illustrating the location of Espírito Santo state and the capital Vitória, Brazil. A—Location of Brazil (blue) within Latin America; B—the Espírito Santo state (green) within Brazil; and C—the city of Vitória (orange) within Espírito Santo state.

Espírito Santo state, located in Brazil's southeastern coastal region, has 78 municipalities and an area of 46 074.448 km^2^ (Figure 1). The state's population in 2022 was 3 833 712 inhabitants [13]. Its climate is tropical‐humid [14]. *A. aegypti* is established in all state municipalities [9]. Vitória is the capital of Espírito Santo state, with an area of 97 123 km^2^, and belongs to the Metropolitan Region of Greater Vitória. In 2022, it had a population of 322 869 inhabitants, with a population density of 3324.33 inhabitants per km^2^ [15]. Vitória was the second municipality in Espírito Santo state in terms of confirmed DENV infections in 2023, with more than 19 000 cases and 6 deaths, and in 2024, with more than 17 000 cases and 2 deaths [8].

### 2.4 Epidemiological Characterization

Dengue epidemiology in Espírito Santo state, Brazil, was described using records from the eSUS Health Surveillance System (eSUS‐VS), the state's electronic platform for mandatory disease notification, maintained by the Espírito Santo State Health Department. The long‐term historical series (January 2007–December 2024) was analyzed by calculating monthly dengue incidence per 100 000 inhabitants to characterize seasonality, identify epidemic periods, and compare the magnitude of recent epidemics with prior cycles, highlighting the timing of peak transmission and contrasting the intensity of 2023–2024 with earlier years in the series.

For the detailed characterization of the most recent period (2020–2024), selected because 2019 was the last major epidemic preceding this period, residents clinically classified as dengue, dengue with warning signs, or severe dengue, confirmed by laboratory or clinical‐epidemiological criteria according to the Brazilian Ministry of Health case definitions and classification criteria, were included [7].

Confirmation criteria, laboratory tests performed among laboratory‐confirmed cases, identified DENV types, hospitalizations, and deaths were described. In addition, other factors were described: the distribution of clinical severity (mild, warning signs, severe), the estimated lethality among all confirmed cases, and, specifically, among dengue with warning signs or severe dengue. Compared proportions across years were performed using Pearson's chi‐square test in Stata v.14.0 (Stata Corp., College Station, TX, USA), with *p* values < 0.05 considered statistically significant. Laboratory confirmation included positive results in the IgM Enzyme‐Linked Immunosorbent Assay (IgM ELISA), reverse transcription polymerase chain reaction (RT‐PCR), detection of the nonstructural protein 1 (NS1), and viral isolation [7].

### 2.5 Sampling Strategy and Inclusion Criteria

A consecutive sampling method was used to collect blood samples from 340 patients aged 18 years or older with suspected dengue fever during the febrile phase (1–4 days after symptom onset) in 2 public health facilities in Vitória between May 2023 and June 2024, with 51 samples collected in 2023 and 289 in 2024. These public health facilities were chosen because they had the highest number of dengue reports in the municipality. Plasma was separated and stored at −80°C until RNA extraction.

### 2.6 RNA Extraction and RT‐qPCR

RNA extraction was performed using the commercial QIAamp Viral RNA Mini Kit (Qiagen) according to the manufacturer's protocols. A starting volume of 140 µL of plasma was used, and the genetic material was eluted in 60 µL of elution buffer. The extracted RNA was stored at −80°C until use. RT‐qPCR was performed using the commercial ZDC molecular assay (Biomanguinhos) kit, enabling the identification of individual infections or coinfections involving the Zika virus (ZIKV), Chikungunya virus (CHIKV), and the four DENV types (DENV‐1, DENV‐2, DENV‐3, and DENV‐4) [16].

### 2.7 Molecular Analysis

To characterize the viral genomes, whole‐genome sequencing was performed as described by Quick and colleagues with some modifications [17]. Only positive samples with a cycle threshold (Ct) < 28 were selected for the next‐generation sequencing, as lower Ct values correspond to higher viral copy numbers (~10^4^ copies per μL or higher), which have been shown to yield near‐complete genome coverage for all four DENV types, using the set of primers previously described elsewhere [18]. The extracted RNA was used for complementary DNA (cDNA) synthesis using the LunaScript RT SuperMix kit (NEB, Cat. E3010). Then, multiplex PCR was performed using primer pools for DENV‐1 and DENV‐2 previously described [18], and the Q5 Hot Start High‐Fidelity 2x MM kit (NEB, Cat. M0494). Samples were normalized, and the library was assembled using NEBNext Ultra II End repair/dA tailing enzymes (NEB, Cat. M7546), followed by ligation of barcodes with the Native Barcoding Kit 96 V14 (ONT, SQK‐NBD 114.96) and the Blunt/TA Ligase Master Mix (NEB, Cat. M0367). Following a purification step with 0.4 AMPure XP beads (Beckman, Cat. A63881), the adapter protein from the Nanopore SQK‐NBD 114.96 kit was ligated. Sequencing was performed using the MinION platform and an R.10.4 chip (ONT).

The sequencing data were generated in POD5 format, and base calling was performed in real time with a high‐accuracy (HAC) setting using MinKNOW Mk1B software (ONT). Reads were trimmed and demultiplexed using MinKNOW. To generate consensus sequences, SeqKit was used to filter small fragments and primers (> 400 bp) [19, 20]. Reads were then mapped against the reference genomes (NC_001477.1 for DENV‐1 and NC_001474.2 for DENV‐2) using Minmap2 and SAMTools [21, 22]. Variant calling was performed with BCFtools, and consensus sequences were constructed using Medaka (Nanoporetech, https://github.com/nanoporetech/medaka) [23].

### 2.8 Phylogenetic Analysis

Of the sequences obtained, only those with greater than 75% coverage were used for phylogenetic analyses to ensure data quality and representativeness. Sequences available in GenBank (www.ncbi.nlm.nih.gov/genbank/) and GISAID (www.gisaid.org) were used to compose the data set for the analysis of each virus. Efforts were made to include genomes from diverse geographic regions of Brazil, as available. Additionally, all 370 DENV genomes from Espírito Santo state (2020–2025) retrieved from GISAID were included in the data set, being 135 DENV‐1 and 97 DENV‐2 (Supporting Information S1: Table 1). The sequences were aligned using MAFFT v.7 [24]. Aliview v.1.28 was used to visually inspect and trim the 5′ and 3′ UTR regions [25]. To identify the genotypes and clades, the alignment was submitted to the Nextclade online platform (https://clades.nextstrain.org/), and the results were input into the metadata table [26]. The maximum likelihood (ML) phylogenetic trees were estimated using the IQ‐TREE v.2.3.6 program [27], with 1000 Bootstrap replicates with UFBoot2 (ultrafast bootstrap) [28] together with 1000 Shimodaira–Hasegawa‐like approximate likelihood ratio test (SH‐aLRT) [29]. The best substitution model was chosen using the ModelFinder, where for DENV‐1, the model was chosen according to the AIC criterion (Akaike's Information Criterion), and for DENV‐2, the best‐fit model was selected by Bayesian Information Criterion (BIC) [30]. The obtained trees were analyzed using FigTree v.1.4.4 [31], and TempEst was employed to verify the temporal signal and identify outlier sequences. Annotated trees were generated in RStudio using the ggplot2 [32] and the ggtree packages [33].

The estimation of the time to the most recent common ancestor (tMRCA) for DENV‐1 and DENV‐2 clades from Espírito Santo state was conducted using Bayesian Markov Chain Monte Carlo (MCMC) methods as implemented in BEAST v1.10.4 [34]. For this analysis, reduced DENV‐1 and DENV‐2 data sets were built from the initial data sets used to construct the ML tree. Sequences with unexpected divergence were identified using TempEst, and those belonging to genotypes outside the target group were excluded from this analysis to ensure phylogenetic consistency, decrease run time, and reduce computational time and memory usage. Analyses employed a Bayesian Skyline (BSL) coalescent framework alongside relaxed, uncorrelated exponential and LogNormal molecular clocks, using time‐stamped data scaled in years and the GTR + I + G nucleotide substitution model. Parameter convergence was assessed with Tracer v1.7.2, and uncertainty was expressed through 95% highest probability density (HPD) intervals.

A total of 100 million MCMC iterations were performed for each data set, with sampling every 10 000 steps. The resulting trees were summarized into a maximum clade credibility (MCC) tree using TreeAnnotator, discarding the first 10% as burn‐in. The MCC tree was then visualized using FigTree v1.4.4.

As an exploratory analysis, the data sets were submitted to Nextclade (https://clades.nextstrain.org) to annotate amino acid substitutions relative to reference sequences for DENV‐1 (NC_001477.1) and DENV‐2 (NC_001474.2) [26]. Substitutions observed in ≥ 50% of Espírito Santo sequences within each lineage are reported descriptively in Supporting Information S2 and S3: Tables 2 and 3. Given that the non‐Espírito Santo comparison group was derived from a phylogenetically subsampled data set rather than a geographically representative sample, no formal statistical comparisons were performed, and the results should be interpreted as hypothesis‐generating rather than conclusive.

## 3 Results

**Figure 2:**
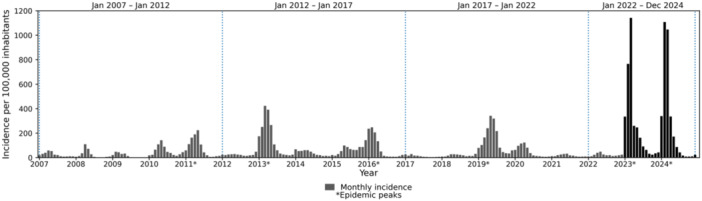
Monthly incidence of dengue cases per 100 000 inhabitants in Espírito Santo state, Brazil, from 2007 to 2024.

Between 2020 and 2024, Espírito Santo state experienced two major dengue epidemics, with the largest occurring in 2024, followed by 2023. In all years, the peak incidence was observed between March and April. These recent epidemics were markedly larger than those observed earlier in the series (2007–2019), when seasonal peaks were recurrent but substantially lower and shorter‐lived. After a period of comparatively low transmission in 2021 (confirmed cases = 8128) and 2022 (confirmed cases = 10 147), incidence rose sharply in 2023 (confirmed cases = 127 070) and remained high into 2024 (confirmed cases = 131 170), with multiple months reaching exceptionally high values, highlighting a substantial increase in epidemic intensity relative to previous cycles (Figure 2).

**Figure 3:**
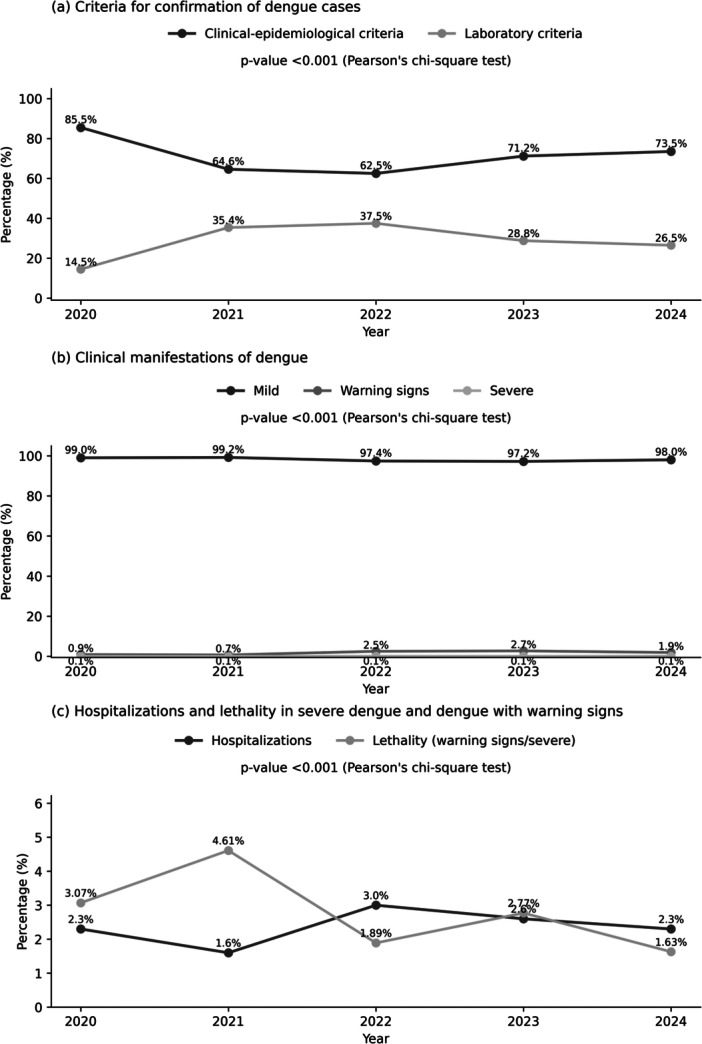
Confirmation criteria (a), clinical manifestations (b), hospitalizations and lethality (c) among confirmed dengue cases in Espírito Santo state, Brazil, 2020–2024.

Across 2020–2024, the profiles of confirmed dengue cases in Espírito Santo state changed significantly, especially during the two largest epidemics (2023–2024), with differences in confirmation methods, clinical manifestations, hospitalizations, and deaths (all *p* values < 0.001) (Figure 3 and Supporting Information S4: Table 4).

Confirmations based on laboratory criteria were lower in 2023 and 2024 (28.8% and 26.5%, respectively) than in 2021 and 2022 (35.4% and 37.5%, respectively), but higher than in 2020 (14.5%). Despite the sharp rise in case counts, the clinical spectrum remained predominantly mild in both epidemic years (97.2% in 2023; 98.0% in 2024); however, dengue with warning signs increased compared with 2020–2021 (2.7% in 2023 and 1.9% in 2024 vs. 0.9% in 2020 and 0.7% in 2021). In 2022, the proportion of dengue with warning signs was 2.5%. In addition, the proportion classified as severe dengue stayed stable at 0.1% throughout the period (Figure 3 and Supporting Information S4: Table 4).

Hospitalizations were relatively stable during the epidemics (2.6% in 2023; 2.3% in 2024), whereas in previous years they ranged from 1.6% in 2021 to 3.0% in 2022. Lethality among dengue patients with warning signs or severe dengue was lower in the epidemic (2.77% in 2023 to 1.63% in 2024) and in 2022 (1.89%) than in 2020 (3.07%) and 2021 (4.61%) (Figure 3 and Supporting Information S4: Table 4).

**Figure 4:**
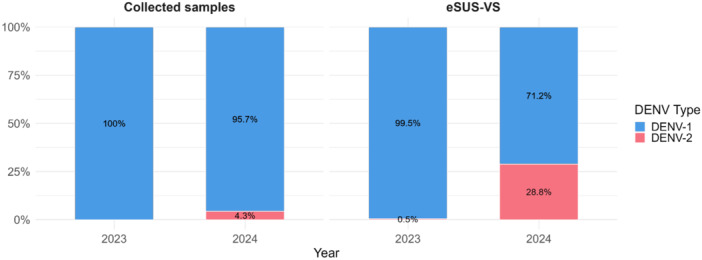
Proportion of dengue virus types 1 and 2 identified in 2023 and 2024 in Espírito Santo state by the samples collected in this study and by the eSUS Health Surveillance System (eSUS‐VS).

The epidemiological surveillance system detected a higher proportion of DENV‐1 throughout the entire period, comprising 93.75% in 2020, 100% in 2021 and 2022, 99.5% in 2023, and 71.20% in 2024. The remaining cases were identified as DENV‐2, while DENV‐3 and DENV‐4 were not detected during this period (Figure 4).

To further investigate the viral diversity underlying the epidemic period, molecular analyses were conducted on clinical samples collected in 2023 and 2024. In 2023, four samples tested positive by RT‐qPCR. Among these, three samples were sequenced, all of which were DENV‐1. In 2024, 51 samples tested positive by RT‐qPCR, and 23 were submitted to sequencing, with 20 belonging to DENV‐1 and 3 to DENV‐2 (Figure 4). Among those, we were able to recover 18 near‐complete genomes (16 DENV‐1 and 2 DENV‐2). A third DENV‐2 genome, previously recovered by our group, was also included, together with DENV genomes available on GISAID and Genbank.

**Figure 5:**
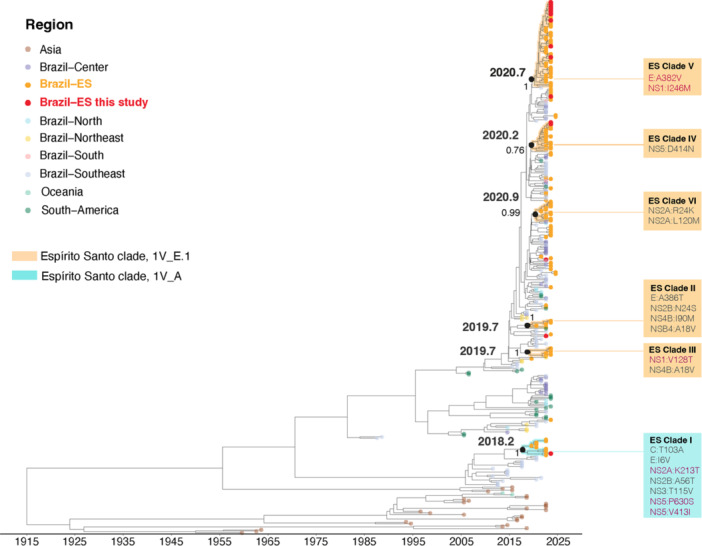
Bayesian time‐calibrated phylogenetic tree of dengue virus type 1 sequences detected in Espírito Santo state, Brazil, 2020–2025. The tree was inferred using BEAST with 349 full‐genome DENV‐1 sequences. Branch lengths are scaled in years. The estimated DENV‐1 introductions into Espírito Santo state are shown, with the lineage 1V_E.1 highlighted in orange and the lineage 1V_A events highlighted in light blue. The corresponding estimated years of the events are shown in bold, along with the posterior probability of the node. Tip colors indicate sampling regions. Brazil‐ES other = sequences from Espírito Santo previously published are shown in orange; Brazil‐ES this study = sequences from Espírito Santo state, this study are shown in red. ES = Espírito Santo. Amino acid substitutions characterizing each Espírito Santo clade relative to non‐Espírito Santo sequences of the same lineage are indicated next to the corresponding node: substitutions in pink are exclusive or near‐exclusive to the Espírito Santo clade (≤ 5% in non‐Espírito Santo sequences of the same lineage); substitutions in gray are enriched in the Espírito Santo clade but also present at lower frequencies in non‐Espírito Santo sequences of the same lineage.

DENV‐1 identified in Espírito Santo state belonged to the genotype V, lineages 1V_A and 1V_E.1 (Figure 5 and Supporting Information S5: Figure 1) [6]. While the cluster formed by Espírito Santo state viruses from lineage 1V_A (ES Clade I, *n* = 11) coalesced in 2018 (TMRCA = 2018.2; 95% HPD = 2017.0‐2019.2), multiple introductions of lineage 1V_E.1 (ES Clades II–VI) were inferred to have occurred between 2019 and 2020 (TMRCA range: 2019.7–2020.9; 95% HPD: 2018.4–2020.6 for ES Clade II, 2019.0–2020.2 for ES Clade III, 2019.8–2020.8 for ES Clade IV, 2020.1–2021.2 for ES Clade V, and 2020.1–2021.5 for ES Clade VI) (Figure 5 and Supporting Information S5: Figure 1). The six larger DENV‐1 clusters from Espírito Santo state were closely related to samples from the Southeast, South, and Central regions of Brazil and South America (Figure 5 and Supporting Information S5: Figure 1). DENV‐1 accumulated genetic mutations and exhibited a consistent, progressive divergence over time, with a clear temporal signal and no significant deviation from the expected evolutionary rate, as observed for strains circulating in other geographical regions (Figure 5 and Supporting Information S5: Figure 1). A root‐to‐tip regression analysis performed in TempEst confirmed a strong positive correlation between sampling date and genetic divergence (*R* = 0.95, *p* < 2.2e − 16), supporting the suitability of the data set for time‐calibrated phylogenetic inference using a molecular clock model (Figure 5 and Supporting Information S5: Figure 1).

All six monophyletic Espírito Santo clades exhibited distinct amino acid substitution profiles, summarized in Figure 5. Within 1V_E.1 (*n* = 139 Espírito Santo sequences across ES Clades II–VI), two substitutions located in functionally important protein domains, E:A382V and NS1:I246M, were detected exclusively within ES Clade V (*n* = 55), where they reached frequencies of 94.5% (52/55) and were absent from the remaining clades (II, III, IV, VI) and from non‐Espírito Santo 1V_E.1 sequences (2.1% and 1.4%, respectively). We also observed broader enrichment of NS2A:S28N (87.1%) and E:K394R (95.7%) in Espírito Santo sequences belonging to 1V_E.1, although the smaller difference relative to non‐Espírito Santo sequences suggests these substitutions are less specific to Espírito Santo circulation and more likely reflect clade‐level background enrichment (Supporting Information S2: Table 2).

Regarding the 1V_A clade sequences, 11 Espírito Santo sequences spanning 2020–2024 were identified, representing a small but temporally diverse subset. Despite the limited sample size, three amino acid substitutions were exclusively observed in Espírito Santo sequences and absent from all 20 non‐Espírito Santo 1V_A sequences in our data set (Brazil, São Paulo and Minas Gerais states, 2012–2022): NS2A:K213T, NS5:P630S, and NS5:V413I. Four additional substitutions (C:T103A, E:I6V, NS2B:A56T, and NS3:T115V) were present in all Espírito Santo sequences but detected in only three non‐Espírito Santo sequences (15%), all from São Paulo state sampled in 2012–2014. These findings suggest that the Espírito Santo 1V_A sequences may represent a distinct sublineage. However, given the small number of sequences available for both groups, these substitutions should be interpreted with caution.

**Figure 6:**
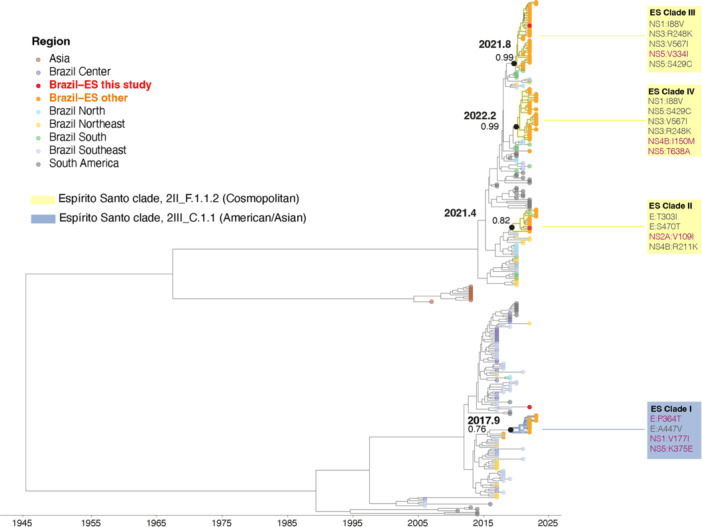
Bayesian time‐calibrated phylogenetic tree of dengue virus type 2 sequences detected in Espírito Santo state, Brazil, 2020–2025. The tree was inferred using BEAST with 307 full‐genome sequences, and branch lengths are scaled in years. Espírito Santo sequences belonging to genotype II, lineage F.1.1.2 (Cosmopolitan, 2II_F.1.1.2) are highlighted in yellow, and those belonging to genotype III, lineage C.1.1 (American/Asian, 2III_C.1.1) are highlighted in dark blue. Four monophyletic groups of Espírito Santo sequences (I–IV) supported by posterior probability ≥ 0.75 are indicated with the corresponding estimated years of the events, which are shown in bold, along with the posterior probability of the node. Tip colors indicate sampling regions. Brazil‐ES other = sequences from Espírito Santo previously published are shown in orange; Brazil‐ES this study = sequences from Espírito Santo state, this study are shown in red. ES = Espírito Santo. Amino acid substitutions characterizing each Espírito Santo clade relative to non‐Espírito Santo sequences of the same lineage are indicated next to the corresponding node: substitutions in pink are exclusive or near‐exclusive to the Espírito Santo clade (≤ 5% in non‐Espírito Santo sequences of the same lineage); substitutions in gray are enriched in the Espírito Santo clade but also present at lower frequencies in non‐Espírito Santo sequences of the same lineage.

The DENV‐2 detected in Espírito Santo state belonged to genotype II (Cosmopolitan), lineage 2II_F.1.1.2, and genotype III (American/Asian), lineage 2III_C.1.1 [6]. DENV‐2 accumulated mutations and diverged consistently and progressively over time, with no notable difference in the expected evolutionary rate compared to strains from other locations (Figure 6 and Supporting Information S6: Figure 2). A root‐to‐tip regression analysis performed in TempEst confirmed a strong positive correlation between sampling date and genetic divergence (*R* = 0.85, *p* < 2.2e − 16), supporting the suitability of the data set for time‐calibrated phylogenetic inference using a molecular clock model (Supporting Information S6: Figure 2).

The Bayesian phylogenetic analysis identified four well‐supported monophyletic clusters of DENV‐2 in Espírito Santo sequences, one belonging to genotype III (American/Asian), lineage 2III_C.1.1 (ES Clade I), and three to genotype II (Cosmopolitan), lineage 2II_F.1.1.2 (ES Clades II–IV) (Figure 6 and Supporting Information S6: Figure 2). The genotype II (Cosmopolitan) clustered with samples from the South, North, and Southeast Brazilian regions, and at least three independent introductions were estimated to have occurred between 2021 and 2022 (TMRCA = 2021.4, 2021.8–2022.2, TMRCA range: 2021.4–2022.2; 95% HPD: 2020.6–2022.4 for ES Clade II, 2021.4–2022.8 for ES Clade III, and 2021.3–2022.3 for ES Clade IV). The DENV‐2 genotype III (American/Asian) clustered with samples from the Brazilian Southeast region, with an estimated introduction in 2017 (TMRCA = 2017.8, 95% HPD: 2017.0–2019.2). DENV‐2 accumulated mutations and diverged consistently and progressively over time, with no notable difference in the expected evolutionary rate compared to strains from other locations (Figure 6 and Supporting Information S6: Figure 2).

The four monophyletic Espírito Santo clades identified in the phylogenetic analysis exhibited distinct amino acid substitution profiles. Within lineage 2II_F.1.1.2 (Cosmopolitan, *n* = 87 Espírito Santo sequences), 4 substitutions were consistently enriched in Espírito Santo relative to 85 non‐Espírito Santo sequences of the same lineage: NS1:I88V (84% in Espírito Santo vs. 34% non‐Espírito Santo), NS3:R248K (79% vs. 35%), NS5:S429C (84% vs. 40%), and NS3:V567I (84% vs. 54%; ES Clades II–IV) (Figure 6). Two additional substitutions were not detected among the non‐Espírito Santo sequences included in this data set: NS5:T638A (36% in Espírito Santo) and NS4B:I150M (13% in Espírito Santo). Notably, NS5:T638A and a second NS5 substitution, NS5:V334I (47% in Espírito Santo, 2% in non‐Espírito Santo), showed mutually exclusive distributions and contrasting temporal dynamics: NS5:V334I was dominant in 2023 (100%) but declined sharply to 10% by 2025, while NS5:T638A was absent in 2023 and rose to 70% by 2025, suggesting an ongoing competitive replacement between two co‐circulating sublineages within clade 2II_F.1.1.2 in Espírito Santo (ES Clades III and IV) (Figure 6). NS4B:I150M mirrored the emergence of NS5:T638A, rising from 0% in 2023 to 40% in 2025, and co‐occurred exclusively with NS5:T638A‐carrying sequences. These two substitutions could define the recently emerging sublineage represented by ES Clade IV (Figure 6 and Supporting Information S3: Table 3).

Within 2III_C.1.1 (American/Asian, *n* = 14 Espírito Santo sequences), 3 substitutions showed strong Espírito Santo enrichment relative to 103 non‐Espírito Santo sequences: NS5:K375E (86% in Espírito Santo vs. 2% non‐Espírito Santo), NS1:V177I (71% vs. 1%), and E:A447V (86% vs. 37%). A fourth substitution, E:P364T, was completely absent from all non‐Espírito Santo 2III_C.1.1 sequences and showed an increasing frequency in Espírito Santo over time, rising from 11% in 2024 to 67% in 2025, suggesting a potential emergence of this variant (ES Clade I) (Figure 6). Although the limited number of 2III_C.1.1 sequences precludes definitive conclusions, the consistent enrichment and temporal increase of these substitutions indicate active adaptive evolution within this clade in Espírito Santo (Figure 6 and Supporting Information S3: Table 3).

## 4 Discussion

During the dengue epidemics of 2023 and 2024, Espírito Santo state experienced its highest incidence of dengue cases in over three decades, with a record‐breaking number of deaths [9]. Both years registered more than 100 000 confirmed cases, resulting in the highest recorded numbers of dengue with warning signs, severe dengue, hospitalizations, and deaths between 2020 and 2024. Case incidence followed a consistent seasonal pattern, peaking approximately 3 months after the maximum temperatures and about 1 month after peak rainfall [35].

The epidemics in 2023 and 2024 occurred in the context of a hyperendemic scenario characterized by the co‐circulation of two lineages of DENV‐1 genotype V and DENV‐2 genotypes II (Cosmopolitan) and III (American/Asian). The co‐circulation of distinct DENV types has been previously linked to large outbreaks [36], raising the hypothesis that the expansion of viral lineages in a susceptible population likely contributed to the magnitude of the epidemics in the state. The simultaneous circulation of distinct DENV was also reported in other regions of Brazil during the same period [10]. The genetic similarity between DENV‐1 and DENV‐2 strains detected in Espírito Santo state and those from other Brazilian states (Southeast, Central, and South) and South American countries indicates both national and intracontinental transmission, likely facilitated by the movement of infected individuals and the transport of vectors across regions [37].

In Brazil, DENV‐1 genotype V was the most prevalent in 2023 and 2024, accounting for more than 80% and 50% of the identified genotypes during the seasonal peaks of those years, respectively [10]. DENV‐1 genotype V, lineage 1V_E.1, was possibly introduced in Espírito Santo state between 2019 and 2020, with some introductions occurring during the COVID‐19 pandemic, while lineage 1V_A was estimated to have been introduced in 2018. The pandemic years (2020 and 2021) were marked by a lethality in dengue with warning signs or severe dengue above 3%, which may indicate an impact on access to healthcare and case management due to concurrent epidemics [38, 39, 40]. This may also have led to underreporting of mild cases, with a relative overrepresentation of severe cases.

The DENV‐1 genotype V lineage 1V_E.1 was the most prevalent lineage in Espírito Santo during the study period, in our sequences and sequences from public databases. This lineage underwent rapid expansion in Espírito Santo, suggesting local diversification consistent with adaptation processes in both human and vector populations [41], culminating in a large‐scale epidemic 4 years after its probable introduction. This lineage was also responsible for causing epidemics in Southern Brazil in 2022 [42]. Data from genomic surveillance in Brazil and other South American countries indicate that lineage 1V_E.1 was frequently detected during the 2023–2024 epidemics, together with other lineages such as 1V_D, also identified in Espírito Santo, and 1V_F, reflecting the diversity of DENV‐1 genotype V circulating in the region [43].

Furthermore, the amino acid substitution analysis of 1V_E.1 sequences within our phylogenetic data set (239 out of 349 sequences) revealed two mutations markedly enriched in Espírito Santo relative to non‐Espírito Santo 1V_E.1 sequences: E:A382V and NS1:I246M. The E:A382V substitution was detected in 56 of 239 1V_E.1 sequences (23.4%), of which 55 originated from Espírito Santo, representing 39.5% of all states' 1V_E.1 sequences in the data set. Similarly, NS1:I246M was identified in 55 of 239 1V_E.1 sequences (23%), with 54 from Espírito Santo (38.8% of all Espírito Santo 1V_E.1). Their frequent co‐occurrence in 54 of 151 Espírito Santo 1V_E.1 sequences, and progressive increase between 2023 and 2024, suggest that local diversification within this lineage is ongoing, potentially reflecting adaptation to locally prevalent immune profiles or vector populations. It is important to note that the E:A382V is located in domain III of the envelope protein, a region involved in host cell receptor binding and targeted by neutralizing antibodies, making this substitution of particular interest for future functional studies [44]. In addition, NS1 mutations at other sites have been associated with modulation of interferon signaling and fitness in the context of antibody‐mediated immune pressure [45].

In Espírito Santo state, the first report of DENV‐1 was in 1996, leading to an epidemic with 39 341 reports in 1998 [9]. However, there is no information on the DENV‐1 genotype identified in the state during this period. DENV‐1 genotype V has been consistently detected in Brazil since the mid‐1990s, with significant introduction events occurring in 1984 and 1985 [46]. Numerous introductions and co‐circulation of multiple lineages of this genotype in the Brazilian territory have contributed to its persistent presence and genetic diversity in the country [47].

Another important finding in our study was the detection of two different DENV‐2 genotypes, co‐circulating in Espírito Santo. Previously, DENV‐2 genotype III (American/Asian) was associated with an epidemic that occurred in 2009 in Espírito Santo state, with 53 179 reports, 393 cases of dengue hemorrhagic fever, 1742 cases of dengue with complications, and 59 deaths [9, 48]. This genotype was possibly reintroduced in Espírito Santo state in 2017, and, in 2019, an epidemic with its predominance accounted for 2324 severe cases and 50 deaths, with a lethality of 2.15 [9]. DENV‐2 genotype III (American/Asian) was detected in Brazil in 1990, with a possible introduction occurring between 1988 and 1989 [48]. It was the only DENV‐2 genotype detected in Brazil up to 2021, when the first report of DENV‐2 genotype II (Cosmopolitan) occurred in Goiás [49].

Despite being one of the DENV genotypes most widely distributed globally, causing epidemics in Asia and Africa, DENV‐2 genotype II (Cosmopolitan) was established in Brazil between 2020 and 2022, with multiple introductions possibly occurring through the border with Peru [49, 50]. Then, it spread rapidly to other Brazilian states and, in 2023 and 2024, was the second‐most prevalent DENV genotype in Brazil [10, 49, 50]. This rapid spread across South America has also been documented in recent studies [51, 52]. Notably, the lineage 2II_F.1.1.2 of this genotype was predominantly detected in genomic surveillance data sets during the 2023–2024 epidemics across Brazil and other South American countries, including Argentina, Bolivia, Paraguay, Peru, and Colombia, representing the most widely dispersed DENV‐2 lineage in the region during this period [43]. The emergence of the DENV‐2 genotype II (Cosmopolitan) in Brazil is epidemiologically significant because it introduces a genetically distinct and globally successful lineage that may alter transmission dynamics and potentially replace the historically dominant DENV‐2 genotype III (American/Asian). Virologically, its divergent evolutionary history and possible fitness advantages warrant close genomic surveillance to assess its impact on dengue spread, population immunity, and future outbreak patterns.

The first report of DENV‐2 genotype II (Cosmopolitan) occurred in Espírito Santo state in February 2023 [49, 50]. However, this strain has been estimated to be circulating in Espírito Santo state at least since the first semester of 2021. This estimate is consistent with the broader national circulation pattern of this genotype, in the Northern region of Brazil, dating to 2021, and from neighboring states such as Minas Gerais, dating to 2022. The absence of DENV‐2 genotype II (Cosmopolitan) sequences from Espírito Santo before 2023 likely reflects the limitations of passive genomic surveillance rather than the true absence of the genotype. Therefore, the increasing severity of cases, hospitalization rates, and lethality among severe dengue cases in 2022 may also be partially influenced by the circulation of this genotype undetected by the health surveillance system.

Moreover, the link between mutations in DENV‐2 proteins, immune‐scape, and pathogenesis has been studied [53]. Within the Espírito Santo DENV‐2 genotype II (Cosmopolitan), in our data set, we identified a temporal shift in the dominant NS5 substitution profile: NS5:V334I predominated in 2023 (10/10, 100%) but was progressively replaced by NS5:T638A, which rose from 0% in 2023 to 32% in 2024 and 79% in 2025. Both substitutions were mutually exclusive across all sequences analyzed. This possible competitive replacement, accompanied by the co‐emergence of NS4B:I150M in the same sequences, suggests that a distinct sublineage within 2II_F.1.1.2 has been gaining fitness advantage in the region, warranting continued genomic monitoring and further investigations to assess its potential impact on viral replication or immune evasion. However, larger data sets would be needed to confirm this pattern.

Mutations in the NS5 protein of DENV‐2 have been shown to accumulate preferentially during intrahost evolution in patients with more severe clinical outcomes, with surface‐exposed substitutions in the RdRp domain potentially modifying protein–protein interactions without impairing core enzymatic activity [53]. These observations underscore NS5 as a functionally plastic locus where adaptive variation may arise during epidemic transmission. Finally, the NS1:K272R substitution, which was recently associated with suppression of STAT1 phosphorylation and interferon‐stimulated gene expression in DENV‐2 [45], was present in 97 of 99 (98.0%) Espírito Santo 2II_F.1.1.2 sequences in our analysis, consistent with the known immune evasion background of this lineage.

The study presents limitations regarding the number of municipalities with DENV sequences and the convenience sampling for genomic analysis. The large scale of the epidemics made comprehensive genomic sampling unfeasible. The use of publicly available sequences from GISAID and GenBank, while broadening the geographic and temporal context of phylogenetic analyses, may not fully capture the diversity of circulating lineages, potentially leading to an underestimation of the number of introduction events or to incomplete resolution of transmission clusters. Moreover, publicly available sequences are predominantly from more populous municipalities, such as the state capital Vitória, leaving smaller and more remote municipalities underrepresented. Beyond genomic limitations, secondary data were used to describe the epidemiological scenario, which is prone to imprecision and under‐ or over‐reporting, especially during the COVID‐19 pandemic [39]. The lower positivity rate in 2023 may have been influenced by the fact that sample collection began after the epidemics peaked in March and April, with the lower incidence affecting the positive predictive value of the clinical diagnosis. Nevertheless, the study captured the complexity of DENV circulation in Espírito Santo state, hypothesizing that the co‐circulation of distinct DENV lineages may have contributed to the unprecedented epidemics of 2023 and 2024.

## 5 Conclusion

The 2023 and 2024 epidemics in Espírito Santo state were associated with the co‐circulation of two DENV‐1 genotype V lineages and two DENV‐2 genotypes, II (Cosmopolitan) and III (American/Asian). This co‐circulation is hypothesized to have contributed to the increased incidence, number of severe cases, hospitalizations, and deaths during this period. The strains were closely related to those found in other Brazilian regions, highlighting regional transmission links, characteristic mutation profiles, and underscoring the importance of continuous genomic surveillance to anticipate and mitigate future epidemics.

## Author Contributions

Conceptualization: Nicolli Ribeiro de Jesus, Camila Malta Romano, and Creuza Rachel Vicente. Methodology: Mariene R. Amorim, Caio Santos de Souza, João Paulo Cola, Camila Malta Romano, and Creuza Rachel Vicente. Formal analysis: Nicolli Ribeiro de Jesus, Mariene R. Amorim, Caio Santos de Souza, João Paulo Cola, Raquel Gomes Catozo, Bruno Luiz M. Guedes, Camila Malta Romano, and Creuza Rachel Vicente. Investigation: Nicolli Ribeiro de Jesus, Mariene R. Amorim, Bruna Caetano Pimenta, João Paulo Cola, Julia Sthefany Nunes Zordan, Yasmim Barcellos Madeira Rosa, Henrique Tamanini Silva Moschen, Isabela Valim Sarmento, and Julia Miranda Fardin. Resources: Aline Souza Areias, Tatiane Comerio, Daniel Claudio de Oliviera Gomes, Camila Malta Romano, and Creuza Rachel Vicente. Data curation: Nicolli Ribeiro de Jesus, Mariene R. Amorim, Caio Santos de Souza, João Paulo Cola, Camila Malta Romano, and Creuza Rachel Vicente. Writing – original draft preparation: Nicolli Ribeiro de Jesus, Mariene R. Amorim, Caio Santos de Souza, João Paulo Cola, Raquel Gomes Catozo, Camila Malta Romano, and Creuza Rachel Vicente. Writing – review and editing: Bruno Luiz M. Guedes, Bruna Caetano Pimenta, Julia Sthefany Nunes Zordan, Yasmim Barcellos Madeira Rosa, Henrique Tamanini Silva Moschen, Aline Souza Areias, Tatiane Comerio, Julia Miranda Fardin, Daniel Claudio de Oliviera Gomes, Kiven Kumar, and Eng Eong Ooi. Visualization: Nicolli Ribeiro de Jesus, Mariene R. Amorim, Caio Santos de Souza, João Paulo Cola, and Raquel Gomes Catozo. Project administration: Camila Malta Romano and Creuza Rachel Vicente. All authors have read and agreed to the published version of the manuscript.

## Ethics Statement

The study was conducted in accordance with the guidelines of the Declaration of Helsinki and was approved by the Research Ethics Committee of the Health Science Center at the Federal University of Espírito Santo (Opinion Numbers: 5.994.874 and 6.241.070).

## Consent

All participants who collected a blood sample signed an informed consent form.

## Conflicts of Interest

The authors declare no conflicts of interest.

## Supporting information


Supporting File 1

Supporting File 2

Supporting File 3

Supporting File 4

Supporting File 5

Supporting File 6

## Data Availability

The epidemiological data supporting the findings of this study are available upon request from the corresponding author. The epidemiological data are not publicly available due to privacy or ethical restrictions. All sequences obtained in this study are available on GISAID (Supporting Information S1: Table 1).
