# Supplementary material for: Genomic and Epidemiological Characterization of DENV‐1 and DENV‐2 Co‐Circulation During the 2023–2024 Dengue Epidemic in Espírito Santo, Brazil
Source: J Med Virol. 2026 Jul 30;98(8):e71078. doi: 10.1002/jmv.71078 (PMC13422009; doi:10.1002/jmv.71078)
Supplement: Supplementary file 4 — Supporting File 4 [file JMV-98-e71078-s001.docx]

**Characterization of confirmed dengue cases among residents of Espírito Santo state from 2020 to 2024**

|  | **2020** | **2021** | **2022** | **2023** | **2024** | **p-value*** |
| --- | --- | --- | --- | --- | --- | --- |
|  | **N (%)** | **N (%)** | **N (%)** | **N (%)** | **N (%)** |  |
| **Confirmed cases** | 27,002 | 8,128 | 10,147 | 127,070 | 131,170 | **<0.001** |
| Clinical-epidemiological criteria | 23,086 (85.5) | 5,253 (64.6) | 6,338 (62.5) | 90,448 (71.2) | 96,419 (73.5) |  |
| Laboratorial criteria* | 3,916 (14.5) | 2,876 (35.4) | 3,809 (37.5) | 36,622 (28.8) | 34,751 (26.5) |  |
| **Clinical manifestations** |  |  |  |  |  | **<0.001** |
| Mild | 26,742 (99.0) | 8,063 (99.2) | 9,883 (97.4) | 123,496 (97.2) | 128,606 (98.0) |  |
| Warning signs | 248 (0.9) | 55 (0.7) | 256 (2.5) | 3,440 (2.7) | 2,456 (1.9) |  |
| Severe | 12 (0.1) | 10 (0.1) | 8 (0.1) | 134 (0.1) | 108 (0.1) |  |
| **Hospitalizations** | 629 (2.3) | 131 (1.6) | 301 (3.0) | 3,287 (2.6) | 3,040 (2.3) | **<0.001** |
| **Deaths** | 8 | 3 | 5 | 99 | 42 | **<0.001** |
| Lethality in confirmed cases | 0.03 | 0.04 | 0.05 | 0.08 | 0.03 |  |
| Lethality in dengue with warning signs or severe dengue | 3.07 | 4.61 | 1.89 | 2.77 | 1.63 |  |

*Pearson’s chi-square test - p-values <0.05 considered statistically significant. 2020: 3,109 IgM, 753 NS1, nine viral isolations, 43 RT-PCR, 3 histopathology, one immunohistochemistry. 2021: 1,617 IgM, 1,265 NS1, 8 viral isolation, 15 RT-PCR, one histopathology, zero immunohistochemistry. 2022: 2,037 IgM, 1,635 NS1, 56 viral isolation, 86 RT-PCR, zero histopathology, two immunohistochemistry. 2023: 15,562 IgM, 18,091 NS1, 511 viral isolation, 6,322 RT-PCR, 47 histopathology, 42 immunohistochemistry. 2024: 10,540 IgM, 18,525 NS1, 127 viral isolation, 13,932 RT-PCR, 431 histopathology, 372 immunohistochemistry. Some patients were submitted for more than one laboratory test.
